# The Impact of Biomass Composition Variability on the Char Features and Yields Resulted through Thermochemical Processes

**DOI:** 10.3390/polym16162334

**Published:** 2024-08-18

**Authors:** Emanuel-Gheorghita Armanu, Marius Sebastian Secula, Bogdan-Marian Tofanica, Irina Volf

**Affiliations:** Faculty of Chemical Engineering and Environmental Protection “Cristofor Simionescu”, “Gheorghe Asachi” Technical University of Iasi, 700050 Iasi, Romania; gheorghita-emanuel.armanu@student.tuiasi.ro (E.-G.A.); marius-sebastian.secula@academic.tuiasi.ro (M.-S.S.); tofanica@tuiasi.ro (B.-M.T.)

**Keywords:** biomass, polymeric components, thermochemical conversion, char, circular economy

## Abstract

This paper explores the intricate relations between biomass polymeric composition, thermochemical conversion routes, char yields and features in order to advance the knowledge on biomass conversion processes and customize them to meet specific requirements. An exhaustive characterization has been performed for three types of biomasses: (i) spruce bark, a woody primary and secondary residue from forestry and wood processing; (ii) wheat straws—agricultural waste harvest from arable and permanent cropland; and (iii) vine shoots, a woody biomass resulting from vineyard waste. Chemical (proximate and ultimate analysis), biochemical, trace elements, and thermal analyses were performed. Also, Fourier transform infrared spectroscopy, Scanning Electron Microscopy, and thermogravimetric analysis were conducted to establish the compositional and structural characteristics of feedstock. The main polymeric components influence the amount and quality of char. The high hemicellulose content recommends wheat straws as a good candidate especially for hydrothermal carbonization. Cellulose is a primary contributor to char formation during pyrolysis, suggesting that vine shoots may yield higher-quality char compared to that converted from wheat straws. It was shown that the char yield can be predicted and is strongly dependent on the polymeric composition. While in the case of spruce bark and wheat straws, lignin has a major contribution in the char formation, cellulose and secondary lignin are main contributors for vine shoots char.

## 1. Introduction

The sustainable utilization of biomass resources is a topic of utmost significance in the dynamic frame of bioeconomy [1]. Biomass plays a pivotal role in the production of valuable bio-based chemicals, materials, and products, shaping a greener circular economy [2]. Including plants, agricultural and forestry waste, wood industry by-products, and other categories, biomass has gained substantial interest also for generating energy and various other applications [3].

The production of lignocellulosic wastes is largely attributed to agricultural and forestry practices among all human activities [4]. Globally, an estimated 140 Gt of biomass waste is produced each year, leading to major environmental impacts and management issues [5].

Around 39.1% of the entire land area in the European Union (EU) is dedicated to agricultural purposes [6], and approximately 23 million tons of dry biomass are produced annually as residual straw from cereals [7]. Moreover, 66% of biomasses are from cereal straw, and more than 60% are generated in low-income countries from the Asian continent and northern African countries [8]. Globally, major crops such as barley, maize, rice, rapeseed, wheat, soybean, sugarcane, and sugar beet produce almost 3.3 Gt of waste (fresh weight) annually [9].

Cellulose and hemicellulose-rich agricultural biomass wastes are valuable feedstocks for producing a wide range of products from chemicals [10], bioactive compounds [11], biomaterials (composites, engineered bioplastics, thermoplastics elastomers, filters and films) [12], supercapacitors [13], carriers for enzyme, bacterial and fungi immobilization [14], amendments [15], and biosorbents [16] to biofuels [17].

Forests cover 38% of land in the EU and provide diverse ecosystem services such as carbon storage and sequestration, habitat provision, air quality and water regulation (quality, quantity, flow), soil erosion control, recreation, and wood and non-wood products [6]. Approximately 70% of forests are composed of two or more tree species, leading to marked variations in forest growth rates. Only a limited part of the biomass from felled trees is removed during harvesting operations, while the main part remains on the ground as primary logging residue [18]. Excessive removal of logging residues from forest sites leads to the depletion of nutrients and organic matter, affecting the soil and, indirectly, altering vegetation and the soil microclimate [19]. Based on Eurostat reports, it is estimated that an average of 281 million tons of trees or tree parts fall each year, of which 224 million tons are removed, while 57 million tons, representing 20%, are left in the forest as logging residues [20]. Consequently, it is imperative to develop a sustainable valorization of biomass waste in order to generate value-added products [21] that can lead to the circular bioeconomy goal [22].

Thermochemical conversion represents a major way to convert biomass waste efficiently using various paths (gasification, pyrolysis, liquefaction, and hydrothermal carbonization) in order to obtain carbon-rich materials with various uses [22].

Gasification is a thermal process where biomass is degraded to hydrogen (H_2_), carbon monoxide (CO), carbon dioxide (CO_2_), and other gasses at high temperatures (600–1200 °C) for a short residence time (10–20 s) [23]. This process demands high energy and also involves high risks of pollution (CO_2_, NO_x_, SO_x_, and solid residues) [24].

Pyrolysis consists of the decomposition of biomass under anoxic conditions at elevated values of temperature (300–650 °C) and pressure (10–80 MPa) [25]. The process leads to biochar, bio-oil, and remnant gasses (CH_4_, CO, CO_2_, and H_2_) [26]. Low values of temperature (slow pyrolysis) and heating rate (0.1–1 °C/s) favor high solid product yield. By contrast, high values of temperature (500–700 °C) and heating rates (10–200 °C/s) increase the carbon percentage and reduce product yield [24].

Hydrothermal liquefaction (HTL) is a process in which biomass macromolecules are hydrolyzed at average temperatures (280–370 °C) and high pressures (5–40 MPa). Under these conditions, water is still in a liquid state (subcritical water) [27]. HTL uses water as a reaction medium [28], and a short retention time for the production of bio-oil [29]. Though HTL is environmentally safe, it still has the drawback of a high energy demand [27]. On the contrary, hydrothermal carbonization (HTC) is a cost-efficient and environmentally friendly method. HTC has the advantage of operating at low temperatures (180–260 °C) and converting biomass in hot liquid (subcritical or supercritical water) to obtain hydrochar without prior drying [30].

An important indicator of biomass conversion efficiency is the char yield [31], which represents the percentage of biomass that remains in the solid carbon phase once the thermal degradation process has been completed [32]. The yield is closely related to the composition and properties of the feedstock [33]. Biomass has a complex polymeric composition (cellulose, hemicellulose, and lignin) and constituents such as extractables and moisture, each with a unique chemical structure and thermal behavior [34]. As a result, the variability in biomass composition plays a significant role in choosing the thermochemical conversion path, as well as in determining the yield and characteristics of the resulting char [35].

Understanding the complex correlation between biomass polymeric composition, the suitable thermochemical process, and char yield and characteristics are crucial for optimizing the conversion process in order to generate tailored materials for various applications [36]. Furthermore, the characteristics of char (porosity, surface area, surface charge, functional groups, and chemical reactivity) can strongly impact its potential applications [37], ranging from energy production [35] and soil amendment [2], uses as biosorbents [38] and carbon sequestration material [39] to carriers for microorganisms [40] and active ingredients [41].

This paper explores the intricate relations between biomass polymeric composition, thermochemical conversion routes, and char yield and features in order to advance the knowledge on biomass conversion processes and their customization to meet specific requirements.

## 2. Materials and Methods

### 2.1. The Biomass Supplies

Three distinct types of biomass, specifically spruce bark (SB), wheat straws (WSs) and vine shoots (VSs), were analyzed. Raw materials were collected from the northeast region of Romania in the autumn season, and dried in an aerated dark room, grinded in a Micro Powder Grinding Mill Retsch GmbH GM 200 (Retsch GmbH, Haan, Germany) with 320 rpm for 20 min. Using a sieve shaker Retsch GmbH AS 200 (Retsch GmbH, Haan, Germany), based on particle size, the feedstock was separated into large (1–2 cm), medium (0.5–1 cm), and small fractions (<0.1 cm) and placed in a desiccator for further use.

### 2.2. Analytical Methods for Biomass Compositional Attributes

The ash content was determined using a calcination furnace (Nabertherm GmbH L3/11/B180, Lilienthal, Germany) at a constant temperature of 550 °C ± 10 °C for 2 h. The C, H, N, and S contents were measured by conducting elemental analysis by means of a Vario Micro elemental analyzer (Elementar Analysensysteme GmbH, Lilienthal, Germany). The oxygen content was then calculated as the residual of the sample following measurement of the aforementioned components and ash content, relative to 100%. To identify, isolate, and purify the major chemical constituents, the protocols outlined in the Technical Association of the Pulp and Paper Industry (TAPPI) standard methods for analysis were followed. These procedures were carried out to assess the chemical composition of SB, WS, and VS [42,43]. The raw material was first extracted with ethanol–benzene (1:2) for 8 h at reflux, then with ethanol for 4 h at reflux, and finally with distilled water at boiling temperature for 1 h (as in TAPPI T204: Solvent Extractives of Wood and Pulp; and TAPPI T264: Preparation of Wood for Chemical Analysis). Samples of extractive-free material were used to determine the lignin, cellulose, and α-cellulose (T203: Alpha-, Beta-, and Gamma-Cellulose in Pulp) contents of the raw materials. The lignin content was determined as both the acid-insoluble (TAPPI T222: Acid-Insoluble Lignin in Wood and Pulp) and acid-soluble lignin, the latter being measured spectrophotometrically at 205 nm using a JASCO V550 spectrophotometer (Jasco International Co. Ltd, Tokyo, Japan). Following this, the holocellulose was quantified by the Jayme–Wise method using ~1 g of raw material. Sample contents of water, ash, and acid-insoluble ash were determined by following the TAPPI standard methods: T211 (Ash in Wood, Pulp, Paper, and Paper-board Combustion at 525 °C) and T244 (Acid-Insoluble Ash in Wood, Pulp, Paper, and Paperboard).

### 2.3. Fourier Transform Infrared Spectroscopy (FTIR) Analysis

All samples were analyzed using a PerkinElmer Spectrum GX1 spectrometer (PerkinElmer LAS GmbH, Rodgau, Germany), equipped with an attenuated total reflection (ATR) crystal of ZnSe (45 degree) accessory for the analysis of solid samples in reflectance mode. For each sample, IR spectra were acquired (64 scans, spectral resolution 4 cm^−1^) in the reflectance mode in the 4000–500 cm^−1^ spectral range, and the average spectrum was reported. To perform the analysis, the samples (of three sample doses: 0.1 g, 0.2 g, and 0.3 g) were pressed onto the crystal. All raw IR spectra were converted into absorbance, baseline corrected, and vector normalized in the same range (Spectrum 10.6.1 software, Perkin-Elmer LAS GmbH, Rodgau, Germany). The above-described spectral parameters of each sample were submitted to Hierarchical Component Analysis (HCA) and Principal Component Analysis (PCA) to evaluate similarities among samples.

### 2.4. Scanning Electron Microscopy (SEM)

In order to obtain morphological, structural, and specific information about the elemental composition (metals and rare elements), the surface was analyzed with a field-emission SEM (Merlin VP Compact, Carl Zeiss, Oberkochen, Germany) in BSE mode coupled to an EDX spectrometer (Bruker Quantax XFlash 5060F, Bruker Nano GmbH, Berlin, Germany), respectively, at an electron acceleration voltage of 11.8 kV and a beam current of about 250 pA. Firstly, the surface was scanned using the in-lens electron detector negatively biased at 958 V to allow for the detection of high-energy back-scattered electrons and completely suppress secondary electrons.

### 2.5. Thermogravimetric Analysis (TGA)

The samples of SB, WS, and VS (~3 mg) were analyzed using a Mettler Toledo TGA/DSC 2 (Mettler Toledo, Columbus, USA) having a heating rate of 20 °C/min, an airflow rate of 60 mL/min, and a temperature accuracy of ±0.2 K. TGA-DTG plots were used to depict the thermal behavior of main polymeric components—hemicelluloses, cellulose, and lignin—and the contents of water and volatile compounds. TGA-DTG curves were plotted using the Origin software (OriginPro 2019b 9.6.5.169 Version).

## 3. Results and Discussion

Spruce bark (SB), wheat straws (WSs), and vine shoots (VSs) are available in large quantities in central and eastern Europe. Spruce bark is a woody primary and secondary residue from forestry and wood processing, wheat straws are an agricultural waste harvested from arable and permanent cropland, and vine shoots are a woody biomass that is a result of waste from vineyards. Spruce bark (SB), a residual material from timber harvesting or fallen trees, presents considerable promise as feedstock. Its fibrous nature renders it well-suited for porous carriers and sequestration carbon materials. Being one of the abundant categories of agricultural waste, wheat straws (WSs) are traditionally used for low-value purposes [35]. One of most unresearched and unused wastes is represented by vine shoots (VSs), a largely available agricultural waste improperly valorized in Europe [7].

### 3.1. Chemical (Proximate and Ultimate Analysis), Biochemical, and Trace Elements Characterization of SB, WS, and VS

In order to propose an appropriate conversion path, and understand the char characteristics and yields, an extensive characterization of the raw biomass in terms of com-position and functions (proximate analysis and ultimate analysis) was carried out. Table 1 presents chemical, biochemical, and trace elements characterization of SB, WS, and VS. 

It can be noted that SB had the highest moisture content at 9.55%, followed by vine shoots at 7.8% and wheat straws at 7.4%. A higher moisture content can affect the efficiency of thermal degradation processes due to the increased energy requirements for water evaporation [44]. These results are in line with data reported by Bejenari et al. (8.35% moisture of dried biomass) [45].

The ash content measured at 600 °C serves as a potential hint of biomass suitability for thermal conversion processes. Ash content primarily consists of inorganic minerals present in biomass, such as silica, potassium, calcium, and magnesium [46]. During thermal conversion, these minerals can contribute to ash formation, which may lead to operational challenges, such as slagging, fouling, and corrosion in combustion or gasification systems [47]. VS exhibited the lowest ash content at 2.1%, indicating a minimal content of inorganics. Following closely, SB had an ash content of 2.75%, whereas WS had the highest ash content at 4%. Geremew et al. [48] found a close value for WS (3.7% ash content). This ranking suggests that among the three biomass sources, due to its low ash content, VSs may constitute a feasible candidate of biomass for thermochemical conversion. 

Variations in hemicellulose, cellulose, and lignin composition significantly influence thermal degradation behavior. VS and SB presented the highest cellulose content, of 49.30% and 48.10%, respectively, while WS had the lowest cellulose content (33.80%). Cellulose is a primary contributor to char formation during pyrolysis, suggesting that VS may yield higher-quality char compared to WS [49].

Carbon content plays a vital role in char formation and yield efficiency during thermal degradation [50]. The carbon content across all biomass types considered in this study is relatively consistent with ash content data, with VSs having the highest value (52.72%), followed closely by SB (52.07%) and WSs (51.56%). Due to their relatively high carbon content, VSs are more likely to yield a higher char amount during thermal conversion processes compared to SB and WS. Therefore, enhancing the efficiency and effectiveness of thermochemical conversion processes, VS represents the most suitable biomass among the three.

Hemicellulose degrades above 125 °C [51]. Among the three types of biomass, WS had the highest concentration of hemicellulose (38.2%), while SB and VS contained significantly lower contents of hemicellulose: 19.7 and 21.5%, respectively. The relatively high hemicellulose content recommends WS as a good candidate for thermochemical conversion processes conducted at low values of temperature and residence time (HTC).

Kalderis et al. [52] reported that cellulose degradation requires temperatures above 200 °C. Following the degradation route, cellulose is hydrolyzed to glucose, and then the dehydration of glucose leads to furfural. The end product is a carbonized structure. Among the three feedstocks, VS had the highest content of cellulose (49.3%), closely followed by SB (48.1%). Thermo-degradation processes conducted above 200 up to 350 °C are expected to generate micro and nanoporous structures in HTC and slow pyrolysis processes, respectively. 

Lignin has a larger span of degradation temperatures, between 200 and 500 °C [53]. Such temperature values of biomass conversion and high contents of lignin (24% for VS, and 22.87% for SB) result in the generation of bio-oil instead of biochar. Consequently, VS and SB are both good candidates for HTC (above 250 °C) and fast pyrolysis.

Ligno-cellulosic content/structures are the major contributors to the porosity, adhesion forces, and multi-layer surface areas of the achieved chars [54].

### 3.2. Fourier Transform Infrared Spectroscopy (FTIR) of Feedstock

**Figure 1:**
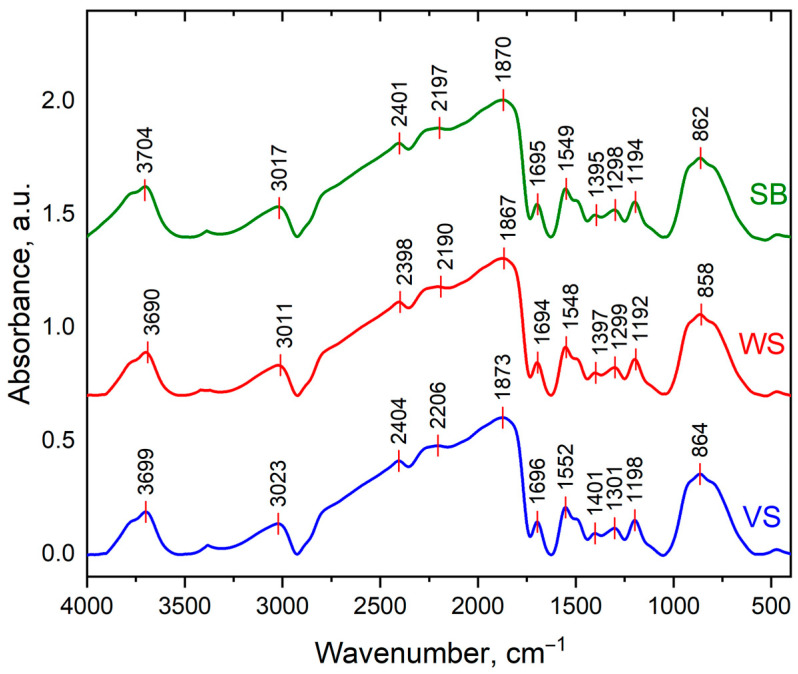
FTIR spectra of SB, WS, and VS, respectively.

The FTIR spectra recorded for all three samples (SB, WS, and VS) were overlapped in order to point out the main different functional characteristics, as shown in Figure 1. The functional groups of each source of biomass are mostly alike, though have several differences, especially in the case of SB and VS.

The band at ≈3700 cm^−1^ is due to stretching vibrations in hydroxyl groups. The position and shape of this band suggest that the hydroxyl groups are involved in hydrogen bonding. The residual water of the raw material could take part in the formation of hydrogen bonds [55]. According to the four types of hydrogen-bonded structures reported previously by Coleman et al. [56], the most predominant are the self-associated -OH groups. Suggesting an aliphatic passage, the presence of C-H and C-O bonds is indicated by the adsorption peaks in the range 2398–3023 cm^−1^. Ascribed to ν (C=O) vibrations in carbonyl groups, the band at 1860 cm^−1^ shows the presence of carbonyl, ester, or carboxyl groups from cellulose and lignin [57].

The ν (C=C) absorptions occur between 1700 and 1400 cm^−1^ [58]. The band at ≈1695 cm^−1^, which is due to olefinic ν (C=C) vibrations, indicates that the olefinic bond is not conjugated with phenyl groups. The aromatic C-C absorptions occur in the 1635–1060 cm^−1^ region. Regarding the bands between 1060 and 830 cm^−1^, the vibrations of C-O-C can be related to asymmetric vibrations in a single graphitic sheet and between two such sheets; oxygen can act as a cross-linking agent between aromatic sheets [55]. Also, some bands are observed in the region >858 cm^−1^, which are band positions compatible with γ(C-H) vibrations in olefinic or aromatic structures.

### 3.3. Scanning Electron Microscopy (SEM) Analysis of Biomass

**Figure 2:**
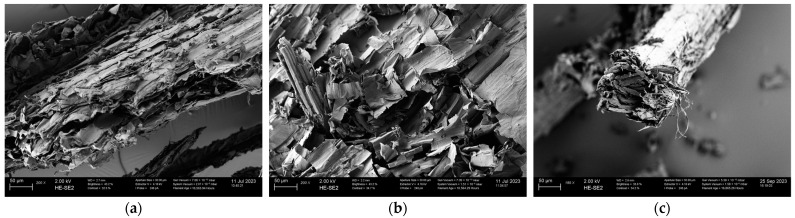
SEM images of the raw biomasses: (**a**) SB; (**b**) WS; (**c**) VS.

The SEM images were recorded in order to investigate the surface morphology of SB, WS, and VS. Figure 2 highlights the common porous structure of the lignocellulosic biomass. Several macropores of various sizes were identified.

The SB (Figure 2a) and WS (Figure 2c) indicate having amorphous shapes with inconsistent porous structures, while VSs (Figure 2b) reveal a scaffold-type structure with long-line micro-fibrils. Another factor that can contribute to the material’s morphology is the grinding process (mechanical deformation) [53].

### 3.4. Thermal Behavior of Feedstock

**Figure 3:**
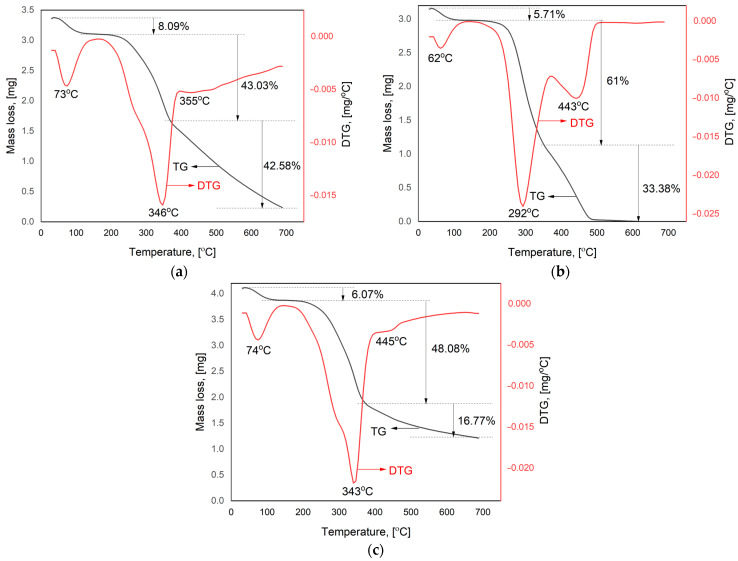
Thermogravimetric analysis of (**a**) SB; (**b**) WS; (**c**) VS.

Figure 3 shows the thermogravimetric analysis (TGA) profiles for the thermal behavior of raw materials. During the thermal decomposition of SB, WS, and VS, three major stages were noticed.

The initial phase of mass loss can be attributed to moisture evaporation. This first phase took place in a temperature range up to 114 °C, displaying a peak centered at 72 °C, for SB; up to 95 °C for WS (peak at 62 °C); and up to 102 °C in the case of VS (peak at 74 °C). The initial weight of the raw materials decreased by 8.09% in the case of SB, 5.71% for WS, and 6.07% for VS. These data support the proximate analysis. 

At temperatures within the range of 255–350 °C, the mass loss is mainly ascribed to the decomposition of the hemicellulose and cellulose components, while lignin is the main polymer component degraded above 350 °C. These results are in line with those reported in [23]. Above 200 °C, the polysaccharide molecules are degraded, resulting in compounds like furans, ketones, carboxylic acids, aldehydes, and phenols [59].

Substantial degradation of SB (43.03%), WS (61%), and VS (48.08%) was observed in the second stage, with temperature peaks at 346 °C, 291 °C, and 343 °C, respectively. The highest degradation percentage was obtained in the case of WS, which had the highest hemicellulose content (38.2%) compared to those of SB and VS (19.75 and 21.5%, respectively). Haykiri-Acma et al. [60] reported that the decomposition of lignin is slow in a wide temperature range due to its complex structure. Nevertheless, this phenomenon is potentially linked to the joint chemical interaction among residual lignin, cellulose, and few inorganic compounds, which partially aligns with the already discussed morphological results [61].

In the third stage, decomposition reached 42.58 in the case of SB, 33.38% for WS, and 16.77% for VS. The third (last) step consisted of the maximum decomposition of the all three materials. All three investigated materials provided degradation rates at certain temperature values according to their chemical composition.

### 3.5. Predicted Yield of Feedstock Conversion

The physical and chemical biomass attributes provide valuable insights for the selection of the most appropriate thermochemical path in order to maximize conversion rates in relation to solid products with high carbon content. To achieve these, a thermal degradation path was developed considering the dataset available for SB, WS, and VS, respectively. 

Theoretical char yields were calculated by leveraging the concentration of main polymeric components. Considering the specific composition of hemicelluloses, cellulose, and lignin in each feedstock, the theoretical calculations serve as a predictive tool, guiding the selection of biomass in order to maximize the char yield (Table 2). A computational procedure based on the practical yields obtained from carbonization processes of pure components was developed [62]. The theoretical yield, *Y* (in %), in the solid phase was calculated using

(1) $$Y\left(wt\%\right)=C\times y_{c,C}+L\times y_{c,L\;}+H\times y_{c,H}$$

where *H*, *C*, and *L* represent the percentages of hemicelluloses, cellulose, and lignin, respectively, while *y_c,j_* denotes the carbonization yield of the main compounds.

Furthermore, an assessment was conducted to determine the role of each polymeric constituent in the generation of char. 

An initial raw amount of 10 g was considered in this study for each type of biomass (SB, WS, and VS). Using Equation (1), the values of the final char amount were calculated, as well as the predicted yield. The hemicellulose/lignin ratio has a significant impact on the formation of water and organics during thermochemical conversion. The presence of hemicellulose can limit the devolatilization of inorganics, increase char formation, and reduce bio-oil yield and quality [63].

After thermochemical conversion, 10 g of SB generates 2.4 g of char, corresponding to a predictive yield of 24.05%. Within the amount of 2.4 g of SB char, 0.46; 0.91; and 1.03 g represent the contribution of main the polymers: *H*, *C*, and *L*, respectively. While in the case of SB and WS, lignin has a major contribution in char formation, cellulose and secondary lignin are main contributors for VS char.

## 4. Conclusions

In this work, an assessment of the main characteristics of three different feedstocks was performed in order to explore the relations between biomass polymeric composition, thermochemical conversion path, and char yield.

It was found that SB has the highest moisture content at 9.55% and therefore requires higher energy input for water evaporation. VS ash content (2.1 wt%) reveals a minimal presence of inorganics and a slow degradation process. Higher inorganic levels (4 wt% in the case of WS) affect the char yield. The carbon content across all biomass types is relatively consistent, with VS having the highest value (52.72 wt%), followed closely by SB (52.07 wt%) and WS (51.56 wt%). Due to the relatively high carbon content, VSs are more likely to yield a higher char amount during thermal conversion processes compared to SB and WS. The relatively high hemicellulose content recommends WS as a good candidate for hydrothermal conversion processes. VS and SB exhibit the highest cellulose content, 49.30 wt% and 48.10 wt%, respectively, while WSs have the lowest cellulose content (33.80 wt%). Due to the fact that cellulose is a primary contributor to char formation during pyrolysis, VS and SB may yield higher-quality char compared to WS.

The FTIR spectra emphasize O-H; C-O-C; C-H; C=C as the main functional groups. The TGA highlights three main degradation stages. This first phase takes place in a temperature range up to 114 °C. The second stage ranges from 255 to 350 °C, where the mass loss is mainly due to the decomposition of the hemicellulose and cellulose components, while the lignin is degraded above 350 °C. The SEM images reveal a common ligno-cellulosic structure: SB and WS have amorphous shapes with inconsistent porous structures, while VS reveals a scaffold-type structure with long-line microfibrils.

It was shown that the char yield can be predicted and is strongly dependent on the biomass polymeric composition. 

This study shows that the composition of biomass determines the adequate thermochemical path and can facilitate the large-scale utilization of agricultural (WS, VS) and forestry residues (SB) for producing value-added materials.

The characteristics of char, including porosity, surface area, surface charge, functional groups, and chemical reactivity can significantly influence its potential applications. These applications encompass a diverse range of areas, including energy production and soil amendment, and they can be used as biosorbents, carbon sequestration materials, and carriers for microorganisms and active ingredients. Future research should continue to explore these relationships, aiming to enhance the efficiency and applicability of biomass-derived char.

## Figures and Tables

**Table 1:** Chemical (proximate and ultimate analysis), biochemical and trace elements characterization of spruce bark, wheat straws, and vine shoots.

Type of Biomass Waste		Spruce Bark	Wheat Straws	Vine Shoots
Proximate analysis	Moisture, wt%	9.55 ± 0.33	7.4 ± 0.28	7.8 ± 0.30
	Ash (600 °C), wt%	2.75 ± 0.29	4 ± 0.34	2.1 ± 0.27
Biochemical components	Extractables, wt%	9.05 ± 0.74	5.1 ± 0.58	2.7 ± 0.45
	Cellulose, wt%	48.10 ± 1.10	33.80 ± 0.92	49.30 ± 1.03
	Hemicellulose, wt%	19.75 ± 0.53	38.20 ± 0.81	21.50 ± 0.57
	Lignin, wt%	22.87 ± 0.48	18.90 ± 0.45	24.40 ± 0.55
Ultimate analysis	Carbon, wt%	52.07	51.56	52.72
	Hydrogen, wt%	5.30	4.49	5.26
	Oxygen, wt%	41.98	43.93	42.01
Trace elements	Copper, ppm	3.1	3.5	3.3
	Cadmium, ppm	1.0	0.1	0.41
	Lead, ppm	2.5	0.2	0.7

**Table 2:** Predicted char yields for SB, WS, and VS.

	Hemicellulose (*H*), g	Cellulose (*C*), g	Lignin (*L*), g	Predicted Yield, %	Predicted Char Weight, g		
					*H*	*C*	*L*
Feedstock	10	10	10	-	-		
Spruce bark	3.38	4.81	4.93	24.05	2.4		
					0.46	0.91	1.03
Wheat straws	1.89	2.30	2.44	25.42	2.5		
					0.5	0.93	1.07
Vine shoots	3.82	1.97	2.15	23.92	2.4		
					0.5	0.98	0.91

## Data Availability

Data available upon request.
